# Is the Determination of Specific IgE against Components Using ISAC 112 a Reproducible Technique?

**DOI:** 10.1371/journal.pone.0088394

**Published:** 2014-02-06

**Authors:** Rubén Martínez-Aranguren, María T. Lizaso, María J. Goikoetxea, Blanca E. García, Paula Cabrera-Freitag, Oswaldo Trellez, María L. Sanz

**Affiliations:** 1 Department of Allergology and Clinical Immunology, Clínica Universidad de Navarra, Pamplona, Spain; 2 Allergology Service, Complejo Hospitalario de Navarra, Pamplona, Spain; 3 Department of Computer Architecture, Universidad de Málaga, Málaga, Spain; University of South Florida College of Medicine, United States of America

## Abstract

**Background:**

The ImmunoCAP ISAC 112 is a fluoro-immunoassay that allows detection of specific IgE to 112 molecular components from 51 allergenic sources. We studied the reliability of this technique intra- and inter- assay, as well as inter-batch- and inter-laboratory-assay.

**Methods:**

Twenty samples were studied, nineteen sera from polysensitized allergic patients, and the technique calibrator provided by the manufacturer (CTR02). We measured the sIgE from CTR02 and three patients' sera ten times in the same and in different assays. Furthermore, all samples were tested in two laboratories and with two batches of ISAC kit. To evaluate the accuracy of ISAC 112, we contrasted the determinations of CTR02 calibrator with their expected values by T Student test. To analyse the precision, we calculated the coefficient of variation (CV) of the 15 allergens that generate the calibration curve, and to analyse the repeatability and the reproducibility, we calculated the intraclass coefficient correlation (ICC) to each allergen.

**Results:**

The results obtained for CTR02 were similar to those expected in 7 of 15 allergens that generate the calibration curve, whereas in 8 allergens the results showed significant differences. The mean CV obtained in the CTR02 determinations was of 9.4%, and the variability of sera from patients was of 22.9%. The agreement in the intra- and inter-assay analysis was very good to 94 allergens and good to one. In the inter-batch analyse, we obtained a very good agreement to 82 allergens, good to 14, moderate to 5 allergens, poor to one, and bad to 1 allergen. In the inter-laboratory analyse, we obtained a very good agreement to 73 allergens, good to 22, moderate to 6 and poor to two allergens.

**Conclusion:**

The allergen microarray immunoassay, ISAC 112, is a repeatable and reproducible in vitro diagnostic tool for determination of sIgE beyond the own laboratory.

## Introduction

Component-based allergological diagnosis has opened up a new era in the study of allergies. It allows the identification of specific sensitization against proteins or specific molecular components [1]–[4]. This new approach helps to clarify the molecular bases of primary sensitization and cross-reactivity phenomena [5]–[11]. It also helps to rationalize the indication for immunotherapy based on the administration of allergenic components [12]–[14] and constitutes a necessary tool in the choice of a diet free from allergens in food-allergic patients [12], [15].

The technique of the commercially available protein microarray ImmunoCAP ISAC® specific IgE (sIgE) 112 offers the possibility of analyzing sIgE against 112 components of purified natural proteins and recombinant proteins from 51 different allergenic sources. Since its launch onto the market, this platform has generated great expectations [16]–[19], and its use is being introduced into clinical practice because, at least from the conceptual point of view, it could be an aid to the clinician in the diagnosis or treatment indication of certain patients, especially those polysensitized ones [6], [20]. However, clinical and technical validations and comparative studies are still needed [21]–[23]. In fact, previous version of this platform (ISAC 103) showed high variability for certain allergens [21], and even low efficiency in diagnosing sensitizations to certain proteins [24]. Thus, data assessing reliability of this technique, now for version ISAC 112, are required even beyond each laboratory.

The objective of this study was to assess the accuracy, precision, repeatability and reproducibility of this platform. To this aim we carried out assays with the technique's calibrating sample and sera from polysensitized patients under different conditions including determinations performed both in the same assay and in different assays and considering as possible sources of additional variability the use of different batches of reagents and the performance of the technique in different laboratories.

## Materials and Methods

### Samples

A total of 20 samples were analyzed: the calibrator sample (CTR02), provided by the manufacturer and 19 sera from polysensitized patients. The research ethical committee from the Universidad de Navarra approved the study “Technical and clinical validation of the diagnostic capacity of microarrays of allergenic molecules in allergy to pollens and/or vegetable foods” in which this work has been done. Patients provided their written informed consent to participate in this study. After inclusion for this work, the data from the patients' sera were analyzed anonymously.

**Figure 1:**
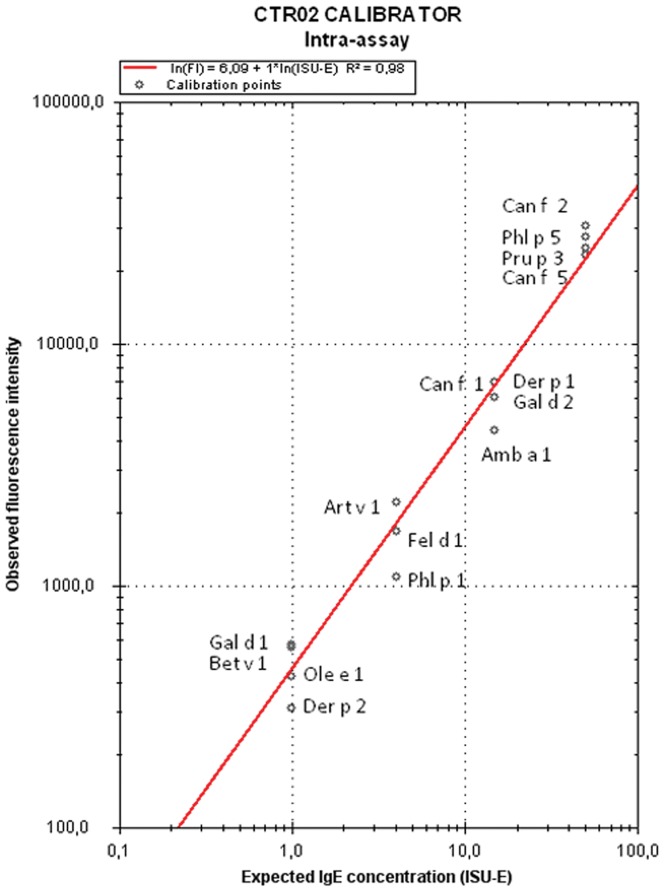
Calibration curve. Example of calibration curve used for the intra-assay analysis.

The CTR02 calibrator, according to information provided by the manufacturer, is composed of known amounts of chimeric monoclonal antibodies humanized against 15 molecular components (Amb a 1, Art v 1, Bet v 1, Can f 1, Can f 2, Can f 5, Der p 1, Der p 2, Fel d 1, Gal d 1, Gal d 2, Ole e 1, Phl p 1, Phl p 5, and Pru p 3). The antibodies against these 15 allergenic components are found in the calibrator in a range of 4 different concentrations expressed in ISAC Standardized Units (ISU) values for sIgE (1, 4, 15 and 50 ISU). This allows a calibration curve to be plotted from the 4 points of fluorescence intensity corresponding to the different ISU (figure 1).

Of the 19 sera from the polysensitized patients, sera 1, 2, and 3, contained as a group detectable amounts of sIgE against the 15 allergens making up the calibration curve and against another 80 allergens (of the 112 represented in the microarray). Sixteen additional sera, numbered 4–19, showing a broad variety of sensitizations were also included in the study.

Patients' sera were frozen at −20°C after the blood collection until the immunoassays were performed and, among the different assays, samples were conserved at 4°C.

### InmunoCAP ISAC 112 microarray

The microarray ISAC 112 (ThermoFisher, Uppsala, Sweden) is a solid phase fluoro immunoassay that detects IgE antibodies against the proteins fixed on ISAC surface. One slide contains 4 microarray and one kit 5 slides, 20 microarrays in total. The technique was performed following the manufacturer's instructions. Each microarray is incubated with a serum in order to label sIgE to each protein and subsequently it is incubated with a human anti IgE detection antibody conjugated with fluorescence.

Finally the fluorescence intensity of each microarray is measured by the scanner (LuxScan 10K/A, CapitalBio, Beijing, China) using following parameters: laser power (LP = 60) and photo-multiplier tube (PMT = 600).

The analysis of the digitalized images is performed with the software Phadia Microarray Image Analyzer (ThermoFisher). This software allows transforming the images fluorescence intensity in numerical data according to the calibration curve built with the calibrator CTR02 sample included in each assay, as previously described (figure 1). An acceptable calibration curve needs to show slope parameters (Y) between 5.5 and 6.8, and R^2^>0.85 according to the information provided by ThermoFisher.

The sIgE values are expressed semiquantitatively as ISU. Results equal or greater than 0.30 ISU are considered positive, according to the indications of the manufacturer.

### Intra-assay analysis

The repeatability of the ISAC 112 technique was assessed by analyzing the sIgE from the CTR02 calibrator and sera 1 to 3 obtained in 10 determinations performed in the same assay with 2 ISAC 112 kits from the same production batch. The calibration curve was obtained with the first determination of the CTR02 calibrator in the first kit in the first slide, first microarray.

### Inter-assay analysis

The reproducibility of the technique was assessed by analyzing the sIgE results from the CTR02 calibrator and sera 1 to 3 obtained in 10 assays performed on 10 different days using 2 ISAC 112 kits from the same production batch. For each assay a calibration curve was plotted with the calibration curve performed in its own assay.

### Inter-laboratory analysis

The reproducibility of the ISAC 112 technique was assessed by analyzing the levels of sIgE obtained from the 20 samples studied with 2 kits from the same batch. The analyses were performed in two different laboratories i.e.: Clínica Universidad de Navarra, and the Complejo Hospitalario de Navarra, both in Pamplona, Navarra, Spain. The reading of the chips was performed with the same conditions in each laboratory. The calibration curve was plotted with the determination of the CTR02 calibrator performed in each laboratory. Both scanners had been calibrated similarly by the same technician, the same day.

### Inter-batch analysis

The reproducibility of the ISAC 112 technique was also assessed by analyzing sIgE levels from the 20 samples under study obtained from two kits from different production batch.

### Statistical analysis of the data

We studied the *accuracy* of ISAC 112, understood as the similarity between the values obtained and those expected, and its *precision*, understood as the dispersion of the group of results obtained.

We evaluated the *accuracy* by the analysis of the determinations performed in all the assays with the sample CTR02, after comparison of the results expected and those obtained for each one of the 15 allergens that constitute the calibration curve by means of the Student's T test.

Furthermore, the *precision* of the allergens from the calibration curve was analyzed by calculating the coefficient of variation (CV) in all the assays, as the percentage of the standard deviation of the determinations divided by the mean. We calculated the CV from the results obtained from the CTR02 calibrator.

We analyzed the strength of agreement of the determinations under the same conditions, *repeatability*, and different conditions, *reproducibility*, of the results obtained in the different assays by calculating the intraclass coefficient correlation (ICC) for each allergen [25], [26]. The level of agreement using the ICC was expressed using the classification of Fleiss [27] as: very good (ICC>0.90), good (0.71–0.90), moderate (0.51–070), mediocre (0.31–0.50) or poor (ICC<0.30).

Also, we analysed the *reliability* of the semiquantitative character of the technique comparing the number of allergens that modified the category of the IgE according to the range established by the manufacturer (<0.3 ISU, not detectable; ≥0.3–<1 ISU, low; ≥1–<15 moderated or high; and ≥15, very high), in the interassay determinations for each serum.

The Student's T test and the ICC for each allergen were performed using the statistical software package SPSS (Statistical Packaged for social science) for Windows, version 15.0 (Chicago, Illinois, USA). The CV was calculated using the program Excel version 12.0, Microsoft Office 2007.

## Results

### Accuracy

**Figure 2:**
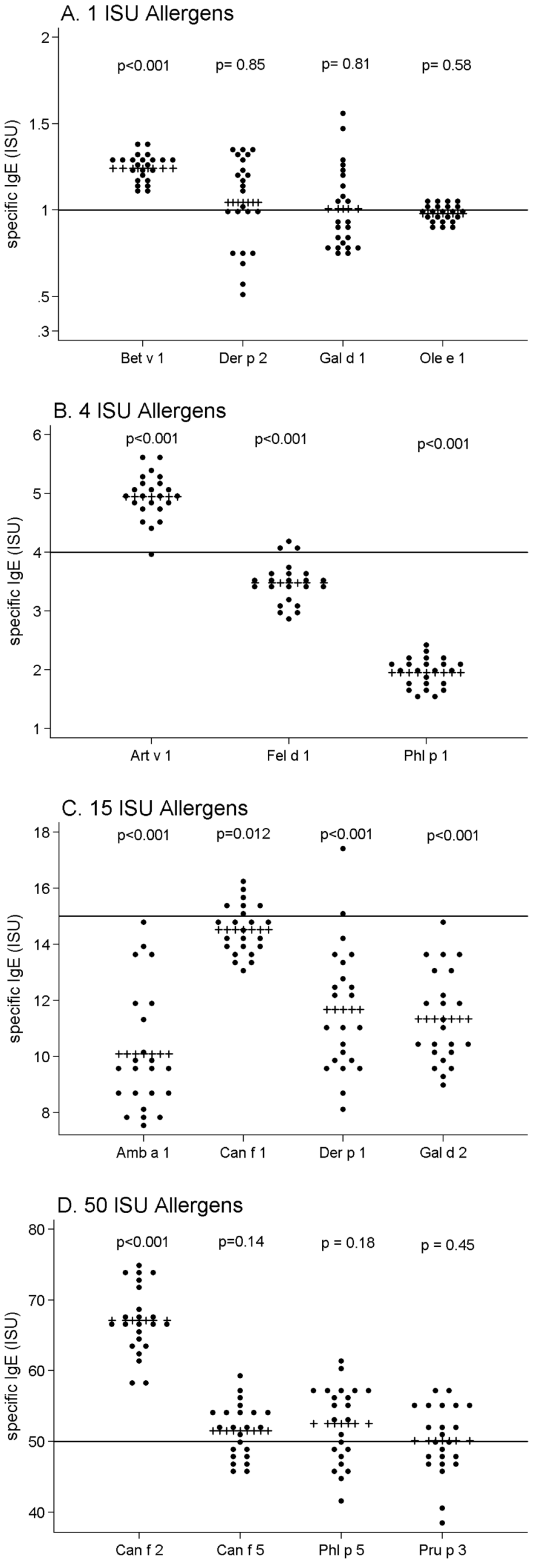
Accuracy of ISAC 112. Results obtained for the allergens that form the calibration curve with the intra-assay, inter-assay, inter-laboratory and inter-batch determinations performed with the calibrator CTR02, composed by chimeric antibodies. Expected ISU results are depicted by red line and the observed mean ISU results are depicted by red +++. Student T test was used for statistical analysis.

The average values obtained in the sIgE determinations against the allergens of the calibration curve Der p 2, Gal d 1, Ole e 1, Can f 1, Can f 5, Phl p 5 and Pru p 3 by ISAC resulted to be similar to those expected (figure 2). Nevertheless, the determinations of the remaining 8 allergens showed statistically significant differences (p<0.05) between the observed levels and those expected. Of these 8 allergens, six allergens (Art v 1, Fel d 1, Phl p 1, Amb a 1, Can f 1, Der p 1 and Gal d 2) are in the intermediate points of the calibration curve (4 and 15 ISU), whereas the Bet v 1 belongs to a 1 ISU calibration point and the Can f 2 belongs to the 50 ISU calibration point. Despite these differences, all the calibrator CTR02 determinations gave place to a calibration curve within the slope parameters and R^2^ considered by the manufacturer as acceptable (Y and R^2^ maximum and minimum obtained at the assays 6.52–5.8 and 0.99–0.95, respectively)

### Precision

When we analyzed the global variability in the results from the different determinations of the CTR02 calibrator, we obtained a mean CV of 9.42%.

**Figure 3:**
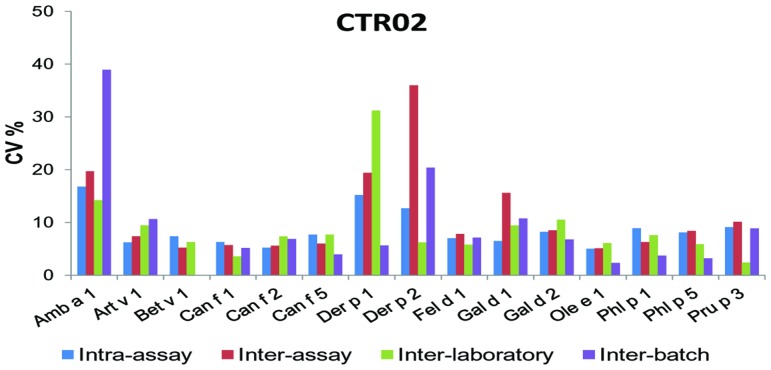
Variability of the ISAC 112 technique in the calibrator sample. Coefficient of Variation (CV) obtained to allergens of calibration curve. The results are the mean CV of 10 determinations intra- and inter-assay, and 2 determinations inter-laboratory and inter-batches of the calibrator sample.

The individual data of the calibrating sera allergens are expressed in figure 3.

### Repeatability and reproducibility

Repeatability and reproducibility calculated using the ICC are summarised in table 1.

**Table 1:** Intraclass Correlation Coefficient (ICC) of ISAC 112 results obtained from 10 determinations of 4 sera intra- and inter-assay and two determinations of 20 sera inter-laboratory and inter-batch.

ALLERGEN	INTRA-ASSAY	INTER-ASSAY	INTER-LAB	INTER-BATCH	ALLERGEN	INTRA-ASSAY	INTER-ASSAY	INTER-LAB	INTER-BATCH
Act d 1	0.983	0.978	0.950	0.995	Equ c 1	0.998	0.983	0.914	0.914
Act d 2	0.999	0.985	0.851	0.879	Equ c 3	0.999	0.996	0.948	0.954
Act d 5	0.978	0.955	0.834	*	Fag e 2	0.998	0.980	0.986	0.996
Act d 8	0.987	0.848	0.907	0.970	Fel d 1 †	0.994	0.987	0.981	0.993
Aln g 1	0.977	0.956	0.867	0.707	Fel d 2	0.996	0.996	0.987	0.775
Alt a 1	0.999	0.993	0.897	0.878	Fel d 4	0.997	0.966	0.970	0.925
Alt a 6	0.988	0.977	0.497	0.267	Gad c 1	0.999	0.986	0.995	0.988
Amb a 1 †	0.996	0.994	0.981	0.900	Gal d 1 †	1.000	0.918	0.970	0.993
Ana o 2	0.998	0.986	0.945	*	Gal d 2 †	0.998	0.969	0.987	0.986
Ani s 1	*	0.994	0.984	0.987	Gal d 3	1.000	0.993	0.998	0.999
Ani s 3	*	*	0.988	0.998	Gal d 5	0.998	0.986	0.997	0.922
Api g 1	0.998	0.978	0.986	0.980	Gly m 4	0.998	0.983	0.998	0.961
Api m 1	*	*	0.973	0.998	Gly m 5	0.997	0.990	0.838	0.944
Api m 4	*	*	*	*	Gly m 6	0.997	0.989	0.955	0.960
Ara h 1	0.997	0.990	0.979	0.963	Hev b 1	*	*	0.945	*
Ara h 2	0.997	0.993	0.937	0.991	Hev b 3	*	*	0.973	0.991
Ara h 3	*	*	0.991	0.928	Hev b 5	1.000	0.995	0.998	0.997
Ara h 6	0.990	0.983	0.868	0.960	Hev b 6.01	0.993	*	0.990	0.995
Ara h 8	0.994	0.980	0.853	0.850	Hev b 8	1.000	0.997	0.999	0.997
Ara h 9	0.997	0.971	0.680	0.992	Jug r 1	0.996	0.986	0.880	0.974
Art v 1†	1.000	0.996	0.703	0.997	Jug r 2	0.994	0.979	0.446	0.881
Art v 3	0.999	0.998	0.986	0.996	Jug r 3	0.999	0.984	0.921	0.983
Asp f 1	0.995	0.984	0.938	0.859	Lep d 2	0.999	0.977	0.941	0.697
Asp f 3	0.999	0.994	0.955	0.998	Mal d 1	0.998	0.994	0.992	0.998
Asp f 6	0.991	0.997	*	*	Mer a 1	1.000	0.996	0.991	0.984
Ber e 1	0.996	0.983	0.779	0.715	Mus m 1	0.992	0.994	0.813	0.997
Bet v 1†	0.999	0.994	0.984	0.973	MUXF3	1.000	0.989	0.923	0.998
Bet v 2	0.999	0.992	0.951	0.946	Ole e 1†	1.000	0.995	0.992	0.990
Bet v 4	0.998	0.990	0.885	0.846	Ole e 7	0.999	0.989	0.831	0.907
Bla g 1	*	*	*	*	Ole e 9	0.999	0.993	0.980	0.995
Bla g 2	*	*	*	*	Par j 2	0.998	0.985	0.998	0.996
Bla g 5	0.992	0.980	0.646	0.776	Pen m 1	*	*	0.994	0.663
Bla g 7	*	*	0.959	0.955	Pen m 2	*	*	*	*
Blo t 5	*	*	0.924	0.993	Pen m 4	0.999	0.995	0.911	0.973
Bos d 4	*	*	0.998	0.969	Phl p 1†	1.000	0.996	0.991	0.989
Bos d 5	0.998	0.995	0.882	0.883	Phl p 11	0.997	0.989	0.994	0.968
Bos d 6	0.999	0.986	0.988	0.983	Phl p 12	0.998	0.979	0.586	0.725
Bos d 8	0.996	0.966	0.566	0.670	Phl p 2	0.998	0.981	0.944	0.886
Bos d Lact	0.865	0.981	0.596	0.464	Phl p 4	0.997	0.993	0.891	0.969
Can f 1 †	0.998	0.993	0.973	0.983	Phl p 5†	1.000	0.992	0.952	0.990
Can f 2 †	1.000	0.999	0.977	0.998	Phl p 6	0.993	0.974	0.829	0.920
Can f 3	0.919	0.981	0.994	0.991	Phl p 7	0.999	0.987	0.784	0.995
Can f 5 †	0.999	0.998	0.996	0.999	Pla a 1	1.000	0.991	0.839	0.818
Che a 1	0.999	0.989	0.940	0.944	Pla a 2	1.000	0.988	0.733	0.904
Cla h 8	*	*	*	*	Pla a 3	0.986	0.964	0.821	0.984
Cor a 1.0101	0.998	0.991	0.998	0.988	Pla l 1	0.990	0.933	0.951	0.994
Cor a 1.0401	0.992	0.984	0.986	0.993	Pol d 5	0.983	0.891	0.996	0.998
Cor a 8	0.999	0.988	0.931	0.990	Pru p 1	0.994	0.944	0.972	0.969
Cor a 9	0.997	0.991	0.827	0.987	Pru p 3†	0.999	0.997	0.985	0.994
Cry j 1	1.000	0.988	0.751	0.839	Sal k 1	1.000	0.997	0.985	1.000
Cup a 1	0.999	0.990	0.981	0.990	Ses i 1	0.990	0.994	0.986	0.533
Cyn d 1	0.999	0.985	0.950	0.909	Tri a 14	0.999	0.993	0.985	0.993
Der f 1	0.999	0.997	0.988	0.944	Tri a 19.0101	1.000	0.994	1.000	0.999
Der f 2	0.991	0.989	0.951	0.955	Tri a aA_TI	0.999	0.994	0.979	0.995
Der p 1 †	0.997	0.994	0.931	0.983	Ves v 5	*	*	0.996	0.634
Der p 10	*	*	0.932	0.994					
Der p 2†	0.993	0.972	0.874	0.961					

* The ICC could not be calculated as all the determinations were equal to 0 ISU.

† Allergens detected by the calibration curve.

ICC ranges: very good (>0.9), good (0.71–0.90), moderate (0.51–0.70), mediocre (0.31–0.50) and poor (<0.30). Underlined are showed ICC values below 0.7.

The analysis of the repeatability (intra-assay analysis) was very good for 94 of the 112 allergens represented in the microarray and good for the Bos d lactoferrin allergen. For the remaining 17 allergens the ICC could not be calculated as all the determinations were equal to 0 ISU.

The analysis of the inter-assay reproducibility was very good for 94 of the 112 allergens and good for the Pol d 5 allergen.

The reproducibility between the analyses of the determinations in two laboratories was very good for 73 allergens and good for 22 of the allergens represented in the microarray. The allergens Ara h 9, Art v 1, Bla g 5, Bos d 8, Bos d lactoferrin, and Phl p 12, showed a moderate agreement whereas in Jug r 2 and Alt a 6 agreement was mediocre. The ICC values could not be calculated for 9 allergens in inter-laboratories analysis because all the determinations for them were equal to 0 ISU.

Finally, in the analysis of the reproducibility between the two kits from different production batches we obtained a very good agreement for 82 allergens and good for 14 allergens. For the allergens Bos d 8, Lep d 2, Pen m 1, Ses i 1 and Ves v 5 we obtained a moderate ICC while for Bos d lactoferrin and Alt a 6 gave a mediocre and poor agreement respectively. The ICC values could not be calculated for 9 allergens in inter-batch analysis because all the determinations for them were equal to 0 ISU.

### Disagreements in the IgE level

When analysing the number of disagreements in the interassay analysis determinations in patients' sera, we obtained the following results. The results of sIgE obtained modified from “not detectable” level to “low” level for 7 allergens when analysing sera 1 and 2, and for 9 allergens when analysing sera 3. The specific IgE levels modified from “low” to “moderate-high” for 7 allergens when analysing sera 1 and 2, and for 13 when analysing sera 3. The specific IgE level change from “moderate-high” to “very high” happened in 16 occasions when analysing sera 1, and in 15 occasions when analysing sera 2. Finally, the IgE level change from “not detectable” to “high” was observed for 4 and 5 allergens when using sera 1 and 2 respectively. This allergens were Act d 1, Asp f 1, Ara h 8, Ber e 1, Cor a 9, Mal d 1, Phl p 5, Phl p 12 and Pla l 1. The allergens that showed a greater number of disagreements were Jug r 2, Jug r 3 and Ses i 1, which had disagreements on the IgE level in the three sera studied.

## Discussion

The technique of protein microarrays has been accepted as a useful method for the detection of sIgE against molecular components [28], [29]. Thus, it has been shown its usefulness for the diagnosis of food allergies, to determine cross-reactivity phenomena or sensitization patterns in specific geographical areas [14], [20]. Previously, several comparative studies between ISAC and other conventional techniques of sIgE detection, such as ImmunoCAP have been performed. These studies state that although the results obtained between both methods are not comparable, a good agreement between them was found [13], [20], [30].

However, few studies have been carried out into the variability and accuracy of this technique showing improvable results in previous versions of this microarray [17], [21]. In the present study, we analyzed accuracy, variability and reproducibility in the new version, ImmunoCAP ISAC 112. The results obtained in the study show good reproducibility of the technique not only for one assay but even considering changes of the assays in different days, and using different batches and in different laboratories.

When analysing the accuracy of the technique, 8 of the 15 allergens that form the calibration curve showed statistically significant differences between the values obtained and those expected. Despite this fact, all the calibration lines were within acceptable limits of slope and R2 established by the technique's supplier. The fact that most allergens keep their category, even at low sIgE levels, supports the reliability of the results offered by the technique. Nevertheless, it should be highlighted that this is a semiquantitative technique and the IgE levels close to the cut off points between categories can fluctuate in different assays.

Regarding the technique's precision, the results obtained show a low variability when we analyse the determinations of the control sample CTR02, which is under 10% in most allergens. Also, the CV is similar both in the analysis performed in the same assay and in those performed in different assays. It is worth noting that the capacity of the allergen to attach itself to the surface of the ISAC array is lower than in quantitative techniques such as ImmunoCAP, in which the allergen is fixed under excess molarity conditions. This fact could make more clear differences in the allergen ability to bind to the microarray leading into differences in variability of specific IgE binding. Differences in reliability of some allergens from the microarray (like Amb a 1, Der p 1 or Der p 2) and considering that ISAC microarray is a semiquantitative technique suggest that ISAC 112 is not the best method to monitor sensitizations and patient's follow-up.

However, in our opinion these analyses demonstrated an evident improvement in the new version of the ISAC microarray. This improvement can be, among others, due to the calibration curve consisting in chimeric antibodies (sIgE) in contrast to the serum with known sIgE concentration from previous versions ISAC 103 [21]. This might be due to a good characterization of chimeric antibodies and the absence of other isotypes able to bind to the spotted allergens.

Finally, we studied the repeatability and reproducibility of the technique, analysing the ICC in the different assays. The results obtained in the intra-assay analysis show a good repeatability, obtaining good agreement strength for almost all the allergens present in microarray. Also, the reproducibility of the interassay analysis showed good concordance strength for these allergens. The reproducibility in the inter-laboratory and inter-batch analysis still has good agreement strength for most allergens. Nevertheless, this agreement strength was lower for 8 allergens in the inter-laboratory analysis and for 7 allergens in the inter-batch agreement. This slight decrease in the results' agreement strength could be due to the fact that only two determinations were performed in each analysis instead of 10 repetitions like in the interassay analysis. Also, the performance of analysis in different laboratories and with different batches means a greater source of variability.

In conclusion, ISAC 112 yields good reliability results taking into account that ISAC 112 gives semi-quantitative results. However, due to the low accuracy obtained in some of the studied allergens, the application of this semi-quantitative technique for diagnosis in clinical situations where results may have a major impact on the therapy prescribed may not be advisable. Even more, this study suggests that neither laboratory specific condition neither the change from one batch to another affect substantially microarray reliability.

## References

[1] Valenta R, Lidholm J, Niederberger V, Hayek B, Kraft D, et al (1999) The recombinant allergen-based concept of component-resolved diagnostics and immunotherapy (CRD and CRIT). Clin Exp Allergy 29: 896–904. 10383589 10.1046/j.1365-2222.1999.00653.x

[2] Asero R, Jimeno L, Barber D (2008) Component-resolved diagnosis of plant food allergy by SPT. Eur Ann Allergy Clin Immunol 40: 115–21. 19227646

[3] Barber D, de la Torre F, Lombardero M, Antépara I, Colas C, et al (2009) Component-resolved diagnosis of pollen allergy based on skin testing with profilin, polcalcin and lipid transfer protein pan-allergens. Clin Exp Allergy 39: 1764–73. 19877313 10.1111/j.1365-2222.2009.03351.x

[4] Lin J, Bardina L, Shreffler WG, Andreae DA, Ge Y, et al (2009) Development of a novel peptide microarray for large-scale epitope mapping of food allergens. J Allergy Clin Immunol 124: 315–22. 19577281 10.1016/j.jaci.2009.05.024 PMC2757036

[5] Gamboa PM, Sanz ML, Lombardero M, Barber D, Sánchez-Monje R, et al (2009) Component-resolved in vitro diagnosis in peach-allergic patients. J Investig Allergol Clin Immunol 19: 13–20. 19274924

[6] Goikoetxea MJ, Cabrera-Freitag P, Sanz ML, Fernández-Benítez M (2010) The importance of in vitro component-resolved diagnosis in paediatric patients. Allergol Immunopathol 38: 37–40. 10.1016/j.aller.2009.11.001 20034723

[7] Palacin A, Quirce S, Armentia A, Fernández-Nieto M, Pacios LF, et al (2007) Wheat lipid transfer protein is a major allergen associated with baker's asthma. J Allergy Clin Immunol 120: 1132–8. 17716720 10.1016/j.jaci.2007.07.008

[8] Barber D, de la Torre F, Feo F, Florido F, Guardia P, et al (2008) Understanding patient sensitization profiles in complex pollen areas: a molecular epidemiological study. Allergy 63: 1550–8. 18925892 10.1111/j.1398-9995.2008.01807.x

[9] Twardosz-Kropfmüller A, Singh MB, Niederberger V, Horak F, Kraft D, et al (2010) Association of allergic patients' phenotypes with IgE reactivity to recombinant pollen marker allergens. Allergy 65: 296–303. 19839972 10.1111/j.1398-9995.2009.02202.x

[10] Díaz-Perales A, Lombardero M, Sánchez-Monge R, García-Selles FJ, Pernas M, et al (2000) Lipid-transfer proteins as potential plant panallergens: cross-reactivity among proteins of Artemisia pollen, Castanea nut and Rosaceae fruits, with different IgE-binding capacities. Clin Exp Allergy 30: 1403–10. 10998016 10.1046/j.1365-2222.2000.00909.x

[11] Ebo DG, Bridts CH, Verweij MM, De Knop KJ, Hagendorens MM, et al (2010) Sensitization profiles in birch pollen-allergic patients with and without oral allergy syndrome to apple: lessons from multiplexed component-resolved allergy diagnosis. Clin Exp Allergy 40: 339–47. 19709127 10.1111/j.1365-2222.2009.03345.x

[12] Ferrer M, Sanz ML, Sastre J, Bartra J, del Cuvillo A, et al (2009) Molecular diagnosis in Allergology: application of the microarray tecnique J Investig Allergol Clin Immunol. 19: 19–24. 19476050

[13] Ott H, Baron JM, Heise R, Ocklenburg C, Stanzel S, et al (2008) Clinical usefulness of microarray-based IgE detection in children with suspected food allergy. Allergy 63: 1521–8. 18925888 10.1111/j.1398-9995.2008.01748.x

[14] Sanz ML, Blázquez AB, Garcia BE (2011) Microarray of allergenic component-based diagnosis in food allergy. Curr Opin Allergy Clin Immunol 11: 204–9. 21522063 10.1097/ACI.0b013e3283466fe4

[15] Renault NK, Mirotti L, Alcocer MJ (2007) Biotechnologies in new high-throughput food allergy tests: why we need them. Biotechnol Lett 29: 333–9. 17160623 10.1007/s10529-006-9251-z

[16] Jahn-Schmid B, Harwanegg C, Hiller R, Bohle B, Ebner C, et al (2003) Allergen microarray: Comparison of microarray using recombinant allergens with conventional diagnostic methods to detect allergen-specific serum immunoglobulin E. Clin Exp Allergy 33: 1443–9. 14519153 10.1046/j.1365-2222.2003.01784.x

[17] Mari A (2001) Multiple pollen sensitization: a molecular approach to the diagnosis. Int Arch Allergy Immunol 125: 57–65. 11385289 10.1159/000053797

[18] Scala E, Alessandri C, Bernardi ML, Ferrara R, Palazzo P, et al (2010) Cross-sectional survey on immunoglobulin E reactivity in 23,077 subjects using an allergenic molecule-based microarray detection system. Clin Exp Allergy 40: 911–921. 20214663 10.1111/j.1365-2222.2010.03470.x

[19] Wöhrl S, Vigl K, Zehetmayer S, Hiller R, Jarisch R, et al (2006) The performance of a component-based allergen-microarray in clinical practice. Allergy 61: 633–9. 16629796 10.1111/j.1398-9995.2006.01078.x

[20] Sastre J, Landivar ME, Ruiz-García M, Andregnette-Rosigno MV, Mahillo I (2012) How molecular diagnosis can change allergen-specific immunotherapy prescription in a complex pollen area. Allergy 67: 709–11. 22379958 10.1111/j.1398-9995.2012.02808.x

[21] Cabrera-Freitag P, Goikoetxea MJ, Gamboa PM, Martínez-Aranguren R, Beorlegui C, et al (2011) A study into the variability of the in vitro component-based microarray technique “ISAC CDR 103”. J Invest Allergol Clin Immunol 21: 414–5. 21905510

[22] Cabrera-Freitag P, Goikoetxea MJ, Beorlegui C, Gamboa PM, Gastaminza G, et al (2011) Can component-based microarray replace fluorescent enzimoimmunoassay in the diagnosis of grass and cypress pollen allergy? Clin Exp Allergy 41: 1440–6. 21749500 10.1111/j.1365-2222.2011.03818.x

[23] Salcedo G, Díaz-Perales A (2010) Component-resolved diagnosis of allergy: more is better? Clin Exp Allergy 40: 836–8. 20557547 10.1111/j.1365-2222.2010.03506.x

[24] Villalta D, Asero R (2010) Is the detection of IgE to multiple Bet v 1-homologous food allergens by means of allergen microarray clinically useful? J Allergy Clin Immunol 125: 1158–61. 20304472 10.1016/j.jaci.2010.01.043

[25] Prieto L, Lamarca L, Casado A (1998) La evaluación de la fiabilidad en las observaciones clínicas: el coeficiente de correlación intraclasse. Med Clin (Barc) 110: 142–45. 9541905

[26] Bravo G, Potvin L (1991) Estimating the reliability of continuos measures with Cronbach's alfa or the intraclass correlation coeficient: toward the integration of two traditions. J Clin Epidemiol 44: 381–390. 2010781 10.1016/0895-4356(91)90076-l

[27] Fleiss JL (1986) Design and Analysis of Clinical Experiments. New York: John Wiley & Sons.

[28] Hiller R, Laffer S, Harwanegg C, Huber M, Schmidt WM, et al (2002) Microarrayed allergen molecules: diagnostics gatekeepers for allergy treatment. FASEB J 16: 414–6. 11790727 10.1096/fj.01-0711fje

[29] Canonica GW, Ansotegui IJ, Pawankar R, Schmid-Grendelmeier P, van Hage M, et al (2013) A WAO - ARIA - GA2LEN consensus document on molecular-based allergy diagnostics. World Allergy Organ J 3 6: 17. 10.1186/1939-4551-6-17 PMC3874689 24090398

[30] Wang J, Godbold JH, Sampson HA (2008) Correlation of serum allergy (IgE) tests performed by different assay systems. J Allergy Clin Immunol 121: 1219–24. 18243289 10.1016/j.jaci.2007.12.1150

